# Knowledge, awareness, and attitudes toward epilepsy in Palestine: a cross-sectional study

**DOI:** 10.3389/fneur.2025.1629227

**Published:** 2025-08-21

**Authors:** Akram Amro, Alhareth M. Amro, Anas K. Assi, Yahya Kayed AbuJwaid, Salahaldeen Deeb, Habeeb H. Awwad, Amro Odeh

**Affiliations:** ^1^Faculty of Health Professions, Al-Quds University, Jerusalem, Palestine; ^2^Faculty of Medicine, Al-Quds University, Jerusalem, Palestine

**Keywords:** epilepsy, knowledge and attitudes, neurological disorders, public awareness, cross-sectional study, Palestine

## Abstract

**Background:**

Epilepsy is a prevalent neurological disorder that remains misunderstood and stigmatized, particularly in resource-constrained settings like Palestine. Misconceptions may hinder diagnosis, treatment, and social inclusion.

**Objective:**

To assess knowledge, awareness, and attitudes toward epilepsy in the Palestinian population and identify sociodemographic predictors.

**Methods:**

A cross-sectional survey was conducted between January and April 2025 using an online, self-administered questionnaire. A total of 570 adults (aged 18–65) participated. Scores for knowledge (0–16), awareness (0–10), and attitude (0–28) were calculated and categorized as “good” or “positive” using predefined cutoffs. Chi-square tests and multivariate logistic regression identified significant predictors.

**Results:**

Among the 570 participants, 297 (52.1%) had good knowledge, 282 (49.5%) had good awareness, and 471 (82.5%) held positive attitudes toward people with epilepsy. Higher education, especially postgraduate, was the strongest predictor of all three outcomes. For example, postgraduate education was associated with significantly greater odds of favorable awareness (OR = 5.60) and positive attitude (OR = 4.38). Male gender was independently associated with lower awareness (OR = 0.59).

**Conclusion:**

While knowledge and awareness remain moderate, attitudes toward people with epilepsy in Palestine are broadly supportive. Educational level is a consistent determinant of improved epilepsy literacy. Targeted public health interventions, especially for men and those with less formal education, can build on existing social acceptance to reduce stigma and promote better care.

## Introduction

Epilepsy is a chronic neurological condition marked by spontaneous and repeated seizures resulting from irregular electrical discharges in the brain. It can affect individuals across all age groups and arises from multiple factors, including genetic traits, brain trauma, developmental issues, or infections (1, 2). According to the World Health Organization (WHO), epilepsy affects over 50 million people globally, presenting a significant health burden, particularly in resource-constrained regions where access to specialized care and public awareness is often lacking (3).

Despite the availability of effective treatments, epilepsy remains misunderstood in many parts of the world. Cultural misconceptions, including beliefs that seizures are caused by spiritual forces, curses, or contagious illnesses, continue to influence how the condition is perceived. These stigmatizing views often lead to social rejection, discrimination, and a reluctance among individuals to disclose their diagnosis or seek medical help, thereby hindering timely care and treatment adherence (4, 5).

Enhancing public understanding is essential to addressing these issues. Educational outreach helps debunk myths, encourages early diagnosis, and equips community members to offer appropriate support during seizures (6). Research in countries such as Ethiopia, Turkey, and Lebanon have highlighted major gaps in public knowledge and negative societal attitudes, underscoring the importance of culturally relevant awareness campaigns (7–9).

In the Palestinian context, epilepsy remains poorly understood. Many individuals are unaware of proper first aid procedures during seizures and continue to associate the condition with mental illness or supernatural causes (10). The ongoing impact of military occupation—through limited healthcare access, disrupted services, and insufficient mental health support—may further contribute to misinformation and stigma (11).

This study aims to evaluate the awareness, knowledge, and attitudes toward epilepsy within the Palestinian population, with the goal of guiding future educational strategies and promoting social inclusion.

## Methodology

### Study design and setting

We conducted a cross-sectional survey between January and April 2025 to assess public knowledge, awareness, and attitudes toward epilepsy in the Palestinian population. Data were collected online using a structured, self-administered questionnaire hosted on Google Forms. Snowball sampling was employed via social media platforms (WhatsApp, Facebook, and Instagram), which, while enabling wide distribution, may introduce selection biases such as homophily bias and reduced researcher control over participant diversity. This method may over represent younger, more educated, and digitally connected individuals.

### Participants

Inclusion criteria were: Palestinian residency, age between 18 and 65 years, and ability to complete an online survey in Arabic or English. We excluded healthcare professionals and students enrolled in healthcare disciplines (medicine, pharmacy, nursing, midwifery, laboratory sciences) to avoid knowledge bias. A minimum sample size of 269 participants was calculated using Epi Info™ (version 7.2), based on an expected awareness proportion of 77.4% drawn from Shawahna’s 2023 study on epilepsy knowledge in Palestine (8), with a 95% confidence level and 5% margin of error. We ultimately enrolled 570 participants to increase statistical power and allow for subgroup analysis.

### Questionnaire development and translation

The survey was adapted from validated tools used by Younes et al. (2024) in Lebanon (9). It comprised five sections: (1) sociodemographic data (age, gender, marital status, education, occupation, income, health insurance, area of residence); (2) medical and social history (chronic conditions, smoking, alcohol use); (3) prior epilepsy education (attendance of lectures/seminars, self-perceived knowledge); (4) knowledge items; and (5) awareness and attitude items. Knowledge items included four binary questions (e.g., “Is epilepsy contagious?”) and four multi-response questions on etiology, clinical manifestations, first-aid measures, and treatment options. Awareness was assessed by five binary items (e.g., having witnessed a seizure, knowing someone with epilepsy). Attitude encompassed 14 statements rated “Yes,” “No,” or “Do not know” (e.g., willingness to work with persons with epilepsy). The questionnaire was forward-translated into Arabic and back-translated to English by independent bilingual experts, with discrepancies reconciled by a third reviewer to ensure semantic and cultural equivalence.

### Scoring and reliability

Responses were numerically coded: for binary and attitude items, “Yes” = 2, “No” = 1, “Do not know” = 0; for multi-response knowledge items, each correct option received 1 point. Total scores ranged 0–16 for knowledge, 0–10 for awareness, and 0–28 for attitude. The cutoffs used to define “good” knowledge (≥9), “good” awareness (≥6), and “positive” attitude (≥17) were adapted from Younes et al. (9) study conducted in Lebanon to ensure comparability with regional data. However, these thresholds have not been formally validated in the Palestinian context. While internal consistency for the attitude (*α* = 0.819) and knowledge (*α* = 0.634) scales was acceptable, the awareness scale demonstrated low reliability (*α* = 0.393), suggesting that the cutoff may be less robust in our sample. Future research should explore context-specific validation of these thresholds using psychometric methods, such as receiver operating characteristic (ROC) curve analysis or item-response theory, to enhance accuracy in classifying participant responses.

### Data management and statistical analysis

Data were exported from Google Forms into SPSS v25 (IBM Corp., Armonk, NY). Descriptive statistics (means ± SD, frequencies, and percentages) summarized participant characteristics and scale scores. Bivariate associations between sociodemographic variables and categorical outcomes (good vs. poor knowledge/awareness; positive vs. negative attitude) were examined using chi-square tests; variables with *p* < 0.20 were entered into multivariate models. Three separate logistic regression analyses identified independent predictors of good knowledge, good awareness, and positive attitude. Adjusted odds ratios (ORs) with 95% confidence intervals (CIs) and *p*-values were reported; statistical significance was set at *p* < 0.05. Model fit was assessed via McFadden’s pseudo-R^2^.

### Ethical approval

The study protocol, including the survey instrument and informed-consent procedure, was reviewed and approved by the Al-Quds University Research Ethics Committee. Participation was voluntary and anonymous; all respondents provided informed consent electronically before accessing the questionnaire.

## Results

A total of 570 participants from the Palestinian population were included in this study. The majority of respondents were female (*n* = 309, 54.2%), and the most represented age group was 18–25 years (*n* = 200, 35.1%), followed by 26–35 years (*n* = 136, 23.9%). Approximately two-thirds of the participants (*n* = 391, 68.6%) held a university degree, while 79 (13.9%) had completed postgraduate studies. Regarding employment status, 144 (25.3%) were employed in the public sector, and 128 (22.5%) were students. Most respondents were non-smokers (*n* = 440, 77.2%), and nearly two-thirds (*n* = 373, 65.4%) had a monthly family income below 6,000₪. Health insurance coverage was reported by 474 participants (83%), with the majority enrolled in governmental or private schemes (Table 1).

**Table 1:** Sociodemographic characteristics of study participants.

Variables basic demographics	*n* (%)
Gender	
Female	309 (54.2%)
Male	261 (45.8%)
Age groups (years)	
18–25	200 (35.1%)
26–35	136 (23.9%)
36–45	122 (21.4%)
46–55	77 (13.5%)
56–65	35 (6.1%)
Educational level	
University	391 (68.6%)
Postgraduate	79 (13.9%)
Secondary	74 (13.0%)
Intermediate	20 (3.5%)
Primary	6 (1.1%)
Employment status	
Public servant	144 (25.3%)
Student	128 (22.5%)
Private-sector employees	102 (17.9%)
Housewife	71 (12.5%)
Freelancer	69 (12.1%)
Unemployed	40 (7.0%)
Retired	16 (2.8%)
Socioeconomic characteristics	
Monthly family income	
3,001–6,000 ₪	187 (32.8%)
1,500–3,000 ₪	186 (32.6%)
Less than 1,500 ₪	101 (17.7%)
More than 6,000 ₪	96 (16.8%)
Health Insurance Coverage	
Governmental	354 (62.1%)
Private	100 (17.5%)
None	97 (17.0%)
UNRWA	19 (3.3%)

Values are expressed as number (percentage).

The analysis of scores revealed that the mean Knowledge Score was 8.5 ± 2.9 (range: 0–16), with 52.1% of participants categorized as having good knowledge. The mean Awareness Score was 5.6 ± 1.8 (range: 0–10), and 49.5% had good awareness. The mean Attitude Score was 20.7 ± 4.9 (range: 0–28), with 82.5% demonstrating a positive attitude toward people with epilepsy. A breakdown of responses to individual attitude statements is presented in Table 2. Most participants supported inclusion in school (91.2%) and work environments (87.6%), while fewer agreed with the right to marry (73.4%) or drive (54.8%). Uncertainty was most common regarding allowing individuals with epilepsy to have children (18.2% selected “Do not know”), reflecting persistent misconceptions and social hesitations. Internal reliability analysis showed a Cronbach’s alpha of 0.819 for the Attitude scale (excellent), 0.634 for the Knowledge scale (acceptable), and 0.393 for the Awareness scale (low).

**Table 2:** Distribution of responses to individual attitude items regarding epilepsy (*n* = 570).

Attitude statement	Yes *n* (%)	No *n* (%)	Do not know *n* (%)
1. Do you think epilepsy patients have the same level of intelligence compared to others?	271 (47.5%)	141 (24.7%)	158 (27.7%)
2. Do you think epileptic patients are crazy or mentally disturbed?	67 (11.8%)	410 (71.9%)	93 (16.3%)
3. Do you think epilepsy patients are more likely to develop mental disorders?	412 (72.3%)	67 (11.8%)	91 (16%)
4. Would you shake hands with an epileptic patient?	508 (89.1%)	24 (4.2%)	38 (6.7%)
5. Would you allow your child to play with a child with epilepsy?	319 (56%)	142 (24.9%)	109 (19.1%)
6. Would you allow your son to marry a girl with epilepsy?	98 (17.2%)	331 (58.1%)	141 (24.7%)
7. Would you allow your daughter to marry a man with epilepsy?	88 (15.4%)	351 (61.6%)	131 (23%)
8. Do you think epilepsy patients can have children?	440 (77.2%)	21 (3.7%)	109 (19.1%)
9. Do you think children with epilepsy have a higher chance of developing birth defects?	129 (22.6%)	204 (35.8%)	237 (41.6%)
10. Do you think epilepsy patients are allowed to drive?	87 (15.3%)	373 (65.4%)	110 (19.3%)
11. Do you think epilepsy patients can do sports?	440 (77.2%)	52 (9.1%)	78 (13.7%)
12. Are you prepared to work with someone with epilepsy?	379 (66.5%)	103 (18.1%)	88 (15.4%)
13. Do you think an epilepsy patient can get a high academic degree?	450 (78.9%)	36 (6.3%)	84 (14.7%)
14. If you were an employer, would you hire someone with epilepsy?	301 (52.8%)	133 (23.3%)	136 (23.9%)

Chi-square analysis indicated a strong relationship between educational level and all three outcome domains. Educational level was significantly associated with knowledge level (*p* = 0.0006), awareness (*p* = 0.0016), and attitude (*p* = 0.0056). No statistically significant associations were observed between knowledge, awareness, or attitude and gender, age, or occupation in bivariate analysis, though some trends were noted in favor of younger participants and those with higher education (Table 3).

**Table 3:** Bivariate associations between sociodemographic factors and knowledge, awareness, and attitude levels toward epilepsy.

Characteristic	Knowledge (*p*)	Awareness (*p*)	Attitude (*p*)
Gender	0.8218	0.1084	0.9707
Age	0.1349	0.4703	0.8367
Marital status	0.7105	0.9695	0.3902
Educational level	0.0006*	0.0016*	0.0056*
Occupation	0.0795	0.1743	0.7288
Smoker	0.4013	0.6009	0.7439
Family income per month	0.2001	0.2904	0.0281*
Health insurance	0.0405*	0.4680	0.2416
Area of residence	0.2731	0.0760	0.1642

*p*-values based on chi-square tests. Asterisks (*) indicate *p* < 0.05.

Multivariate logistic regression revealed several important independent predictors. In the Awareness model, being male was associated with lower awareness (OR = 0.59; 95% CI: 0.40–0.88; *p* = 0.0103). In contrast, university education (OR = 2.82; 95% CI: 1.05–7.59; *p* = 0.0402) and postgraduate education (OR = 5.60; 95% CI: 1.79–17.55; *p* = 0.0031) were associated with significantly higher awareness. In the Attitude model, postgraduate education was a strong positive predictor (OR = 4.38; 95% CI: 1.41–13.65; *p* = 0.0107). Full results of these significant associations are summarized in Table 4.

**Table 4:** Multivariate logistic regression: significant predictors of good awareness and positive attitudes toward epilepsy.

Model	Predictor	OR	95% CI	*p*-value
Awareness	Gender male*	0.59	(0.40–0.88)	0.0103
Awareness	Educational level university*	2.82	(1.05–7.59)	0.0402
Awareness	Educational level postgraduate*	5.60	(1.79–17.55)	0.0031*
Attitude	Educational level postgraduate*	4.38	(1.41–13.65)	0.0107*

Odds ratios (ORs) with 95% confidence intervals (CI) are shown. Asterisks (*) indicates statistical significance (*p* < 0.05).

A focused logistic regression model using Knowledge Level as the sole dependent variable was also conducted. While none of the predictors reached statistical significance, a trend was observed among participants aged 26–35 years (OR = 1.53; 95% CI: 0.82–2.85; *p* = 0.1818). Male gender was associated with lower odds of good knowledge (OR = 0.79; *p* = 0.2382), but the result was not significant. Full details are presented in Table 5.

**Table 5:** Logistic regression analysis of factors associated with good knowledge about epilepsy among participants.

Variable	OR (95% CI)	*p*-value
Constant	1.08 (0.33–3.52)	0.8960
Gender male	0.79 (0.53–1.17)	0.2382
Age 26–35	1.53 (0.82–2.85)	0.1818
Age 36–45	1.11 (0.58–2.13)	0.7449
Age 46–55	0.74 (0.36–1.54)	0.4208
Age 56–65	0.96 (0.35–2.60)	0.9352
Educational level postgraduate	2.62 (0.87–7.83)	0.0857
Educational level primary	0.17 (0.02–1.80)	0.1413
Educational level secondary	0.77 (0.28–2.15)	0.6181
Educational level university	1.48 (0.57–3.81)	0.4192
Occupation housewife	0.86 (0.40–1.86)	0.7081
Occupation private-sector employees	1.73 (0.88–3.39)	0.1108
Occupation public servant	0.94 (0.49–1.79)	0.8394
Occupation retired	1.44 (0.36–5.74)	0.6016
Occupation student	1.17 (0.56–2.44)	0.6834
Occupation unemployed	0.69 (0.31–1.57)	0.3810

Logistic regression examining predictors of good knowledge level. OR, odds ratio; CI, confidence interval. *p*-values <0.05 are considered statistically significant.

Finally, a comparative regression table summarizing predictors across all three outcomes Knowledge, Awareness, and Attitude was developed. This table highlights that education level, especially at the postgraduate level, is a consistently strong and significant predictor across all models. Meanwhile, male gender remains a risk factor for lower awareness (Table 6).

**Table 6:** Factors associated with knowledge, awareness, and attitude toward epilepsy among participants.

Variable	Knowledge OR (95% CI)	Knowledge *p*-value	Awareness OR (95% CI)	Awareness *p*-value	Attitude OR (95% CI)	Attitude *p*-value
Gender (male vs. female)	0.79 (0.53–1.17)	0.2382	0.59 (0.40–0.88)	0.0103	0.85 (0.56–1.28)	0.4333
Age 26–35 (vs. 18–25)	1.53 (0.82–2.85)	0.1818	0.56 (0.29–1.05)	0.0705	0.75 (0.39–1.47)	0.4072
Age 36–45 (vs. 18–25)	1.11 (0.58–2.13)	0.7449	0.58 (0.30–1.15)	0.1197	0.66 (0.33–1.33)	0.2451
Age 46–55 (vs. 18–25)	0.74 (0.36–1.54)	0.4208	1.05 (0.48–2.30)	0.9097	0.79 (0.36–1.77)	0.5721
Age 56–65 (vs. 18–25)	0.96 (0.35–2.60)	0.9352	0.50 (0.18–1.40)	0.1866	0.71 (0.25–2.01)	0.5154
Education: postgraduate	2.62 (0.87–7.83)	0.0857	5.60 (1.79–17.55)	0.0031	4.38 (1.41–13.65)	0.0107
Education: primary	0.17 (0.02–1.80)	0.1413	3.23 (0.45–23.30)	0.2457	1.62 (0.23–11.34)	0.6245
Education: secondary	0.77 (0.28–2.15)	0.6181	1.90 (0.65–5.50)	0.238	1.00 (0.36–2.81)	0.9956
Education: university	1.48 (0.57–3.81)	0.4192	2.82 (1.05–7.59)	0.0402	2.16 (0.83–5.65)	0.1165
Occupation: housewife	0.86 (0.40–1.86)	0.7081	0.79 (0.37–1.71)	0.5538	0.57 (0.25–1.29)	0.1755
Occupation: private sector	1.73 (0.88–3.39)	0.1108	1.63 (0.83–3.20)	0.154	0.63 (0.30–1.31)	0.2145
Occupation: public servant	0.94 (0.49–1.79)	0.8394	1.05 (0.54–2.02)	0.8848	0.53 (0.26–1.09)	0.0827
Occupation: retired	1.44 (0.36–5.74)	0.6016	2.74 (0.58–12.96)	0.2033	0.69 (0.16–3.03)	0.6228
Occupation: student	1.17 (0.56–2.44)	0.6834	0.76 (0.35–1.63)	0.4818	0.61 (0.26–1.41)	0.2465
Occupation: unemployed	0.69 (0.31–1.57)	0.381	1.11 (0.48–2.57)	0.816	0.60 (0.24–1.49)	0.2702

Logistic regression results showing odds ratios (OR) with 95% confidence intervals (CI) and *p*-values. Significant results (*p* < 0.05) are indicated with an asterisk (*).

## Discussion

This study provides a comprehensive overview of public knowledge, awareness, and attitudes toward epilepsy in the Palestinian population. The findings reveal a nuanced picture: while just over half of participants demonstrated good knowledge (52.1%; mean score 8.5 ± 2.9) and awareness (49.5%; mean score 5.6 ± 1.8) of epilepsy, an overwhelming majority (82.5%; mean score 20.7 ± 4.9) exhibited a positive attitude toward individuals living with the condition. Educational level, particularly at the postgraduate level, emerged as the most consistent and significant predictor across all three outcome domains. Notably, male gender was associated with lower awareness levels (OR = 0.59; 95% CI: 0.40–0.88; *p* = 0.0103), highlighting a gender disparity that warrants attention.

These findings are partially consistent with regional and international studies. A 2023 Lebanese survey reported significantly higher rates of good knowledge (87.4%) and awareness (70.1%), though the proportion of participants with positive attitudes (88%) closely matched our findings (8). The discrepancy in knowledge and awareness may reflect differences in public health infrastructure, the reach of continuing education programs, or cultural factors influencing information dissemination. Although knowledge levels were moderate among our participants, most participants correctly identified that epilepsy is not contagious and recognized key clinical features. However, less than one-quarter knew it is incurable, highlighting specific misconceptions that warrant targeted educational interventions.

Other countries in the Middle East show varied patterns. A 2022 Jordanian study found that only 35.3% of participants had good knowledge of epilepsy, although 63.3% reported favorable attitudes (12). In 2021, a systematic review conducted in Saudi Arabia highlighted that persistent cultural misconceptions were evident; over half of the respondents attributed epilepsy to supernatural causes such as jinn, and only 30% correctly identified first-aid measures during a seizure (13). In comparison, the Palestinian population exhibited comparatively better medical knowledge and more supportive social attitudes, despite enduring cultural myths.

In 2023, a study in Bahrain revealed a very high public awareness (95.6%), but stigma remained a concern. For example, 27.5% of participants opposed marriage with individuals who have epilepsy (14). In contrast, Palestinian participants, while exhibiting lower awareness levels, showed greater acceptance and positive attitudes. This contrast underscores the importance of evaluating both cognitive understanding and social perceptions in assessing epilepsy literacy.

Outside the MENA region, similar trends are observed. A 2007 study in Turkey reported moderate public awareness, with 45.2% showing good knowledge and 55.5% demonstrating positive attitudes (15). In Cameroon’s Akwaya District, a 2009 study revealed high levels of misinformation; 25.5% believed epilepsy was contagious, and 33.3% viewed it as a form of mental illness. Moreover, 38.1% felt people with epilepsy should not marry, and 28.6% believed they should not have children. Education again emerged as a strong predictor of better knowledge and attitudes (16). Compared to these contexts, the Palestinian population reflected better outcomes in both knowledge and attitude, albeit with continued misconceptions among certain subgroups.

A 2024 study of Chinese university students found nearly universal awareness (94.7%), especially among medical students, and high levels of accurate first-aid knowledge (16). In contrast, in 2023 a meta-analysis of Ethiopian studies reported that only about 47% of respondents demonstrated good knowledge and a similarly low rate of favorable attitudes (7). These disparities demonstrate the significant influence of healthcare resources, educational infrastructure, and cultural beliefs on public perceptions of epilepsy across different regions.

Educational level was the single strongest determinant across all domains in our study. Participants with postgraduate education were more than five times as likely to have good awareness (OR = 5.60; 95% CI: 1.79–17.55; *p* = 0.0031) and nearly five times as likely to hold positive attitudes (OR = 4.38; 95% CI: 1.41–13.65; *p* = 0.0107) compared to those without formal higher education. University graduates also had over double the odds of good awareness (OR = 2.82; 95% CI: 1.05–7.59; *p* = 0.0402). This trend is mirrored in several international studies. In Lebanon, widespread health education initiatives were associated with higher knowledge and awareness levels (8). Similarly, the Chinese study highlighted the role of academic exposure, particularly in health-related fields, in shaping informed perspectives (16). Conversely, countries with limited educational outreach, such as Jordan (12), demonstrated lower levels of epilepsy knowledge.

Cultural beliefs also play a crucial role. In Saudi Arabia, traditional explanations such as jinn possession persist despite medical advances (13). Bahrain, although more medically informed, still reported significant stigma (14). These findings emphasize that improving knowledge does not automatically eliminate negative attitudes. Interestingly, our study found the reverse trend. Although public knowledge levels were moderate, the population showed a remarkably empathetic and supportive attitude toward individuals with epilepsy, suggesting that social norms in Palestine may be shifting toward greater inclusivity. Furthermore, nearly half of respondents still believed that people with epilepsy should not drive a view that, while sometimes clinically justified, may also reflect lingering stigma. Nevertheless, 75% of participants supported full social integration in employment and marriage, aligning with broader regional improvements in public attitudes.

In the Palestinian context, Palestinian folk beliefs often frame epilepsy in supernatural terms. Classic ethnographies note that seizures are attributed to evil spirits or other demonic forces. For example, Tawfiq Canaan’s studies describe epilepsy as “an illness inflicted by the evil spirits” (17). These ideas coexist with religious interpretations, and many Palestinians view epilepsy as divine punishment for sin or moral transgression (17). Even in modern surveys, small but notable minorities cite spiritual causes. A recent study of medical students in the West Bank found that 7–8% still believed epilepsy is caused by jinn, and 5–6% invoked the “evil eye” or divine punishment as causes (18). These traditional beliefs feed powerful stigma. As the WHO notes for the Eastern Mediterranean region, “stigma and discrimination dominate social attitudes toward epilepsy” (19), Epilepsy is widely feared and misunderstood. Families may hide a diagnosis, and sufferers can be socially excluded or viewed as contagious or cursed. For example, community attitudes often leave people with epilepsy ostracized or unfit for marriage (19). This stigma is reinforced by legal and social barriers. In many Palestinian and neighboring communities, epilepsy carries official restrictions and pervasive taboo. This cultural framing becomes a barrier to social inclusion. In practice, people with epilepsy often face discrimination in schooling, marriage, and employment due to these misconceptions. Indeed, driving and work limitations linked to the condition have been documented as major challenges in Eastern Mediterranean countries (19). Overall, cultural misperceptions and fear add a “vicious circle” of stigma: the affected person is marginalized, which in turn reinforces negative attitudes (19). These beliefs can both hinder and facilitate treatment depending on the community’s trust in biomedical care.

However, Palestine also has strong social support structures based on extended family and religious obligation, which may contribute to the overwhelmingly positive attitudes seen in this study despite moderate knowledge levels (19). Community-centered initiatives that involve mosques, local leaders, and trusted educators may be particularly effective in addressing misconceptions and improving seizure first-aid literacy. Prior research in Palestine has similarly found that integrating health messaging with religious or cultural narratives can reduce stigma and promote early health-seeking behavior (8). Future campaigns should tailor their messaging to these local dynamics for greater impact (10).

This study has several strengths. It is among the first large-scale surveys to assess epilepsy-related knowledge, awareness, and attitudes in Palestine. The use of a validated questionnaire allows for meaningful comparison with international data. The sample size was robust, and internal reliability metrics were within acceptable ranges for most scales—acceptable for knowledge (*α* = 0.634) and excellent for attitude (*α* = 0.819). However, the awareness scale demonstrated low internal consistency (Cronbach’s *α* = 0.393), which may reflect the limited number and binary structure of its items. This weak reliability may reduce confidence in awareness-related findings and suggests that the scale items may not have consistently reflected a unified dimension of awareness. Additionally, given the high proportion of participants reporting favorable attitudes toward people with epilepsy (82.5%), the possibility of epilepsy (82.5%), social desirability bias must be considered. Participants may have been inclined to select responses that align with socially accepted norms, particularly in an online, self-administered format where anonymity may not fully mitigate impression management. This could lead to an overestimation of positive attitudes and mask more nuanced or ambivalent views. To address these issues, future research must expand the awareness scale with more items and transition from binary to Likert-type or multi-response formats to capture nuanced understanding, coupled with rigorous psychometric development processes including pilot testing, item analysis, and factor analysis. Concurrently, comprehensive strategies to mitigate social desirability bias are essential, such as enhancing anonymity, employing indirect questioning techniques, incorporating social desirability scales, and adopting more representative sampling methods, all aimed at ensuring more robust, accurate, and generalizable insights into epilepsy perceptions in Palestine (8). A notable limitation of this study is the use of cutoffs derived from a Lebanese population without formal validation in the Palestinian context. While we adopted established cutoffs from a culturally similar regional study to enable meaningful cross-regional comparisons, these thresholds may not optimally reflect the Palestinian population’s characteristics. However, several factors support the appropriateness of this approach: the Lebanese study used identical questionnaire items, the populations share similar cultural and linguistic backgrounds, and the percentage-based rationale (56–60% threshold) provides a reasonable standard for defining adequate performance.

The chosen cutoffs demonstrated practical utility in our analysis, effectively differentiating between participants with varying educational levels and producing meaningful statistical associations. Educational level emerged as a consistent predictor across all domains, suggesting that the cutoffs successfully captured genuine differences in epilepsy literacy. Additionally, the resulting distributions (52.1% with good knowledge, 49.5% with good awareness, and 82.5% with positive attitudes) appear reasonable and align with expected patterns in the literature.

Nevertheless, future research should establish Palestinian-specific cutoffs through comprehensive validation studies, potentially using criterion-referenced approaches with clinical or behavioral outcomes, expert panel consensus, or receiver operating characteristic (ROC) analysis to optimize threshold selection for the local context.

The study’s reliance on online snowball sampling introduced notable limitations, significantly impacting the representativeness and generalizability of the findings. This method, while facilitating wide distribution, inherently leads to selection biases, including homophily bias (where participants recruit others similar to themselves) and social network bias, which can lead to overrepresentation of certain demographic groups; particularly younger and more educated individuals. Additionally, the lack of control over referral chains reduced researcher oversight of sample structure. Future research should employ stratified or random community-based sampling to improve representativeness and generalizability. Additionally, the wide confidence intervals observed in some regression estimates suggest limited statistical power for certain subgroups, possibly due to small sample sizes within those categories. As a result, the findings may not fully capture the perceptions of older, rural, or less-educated populations. Accordingly, the observed associations between demographic factors and outcomes (knowledge, awareness, and attitudes) should be interpreted as correlations rather than causal relationships. Future studies should consider employing more representative sampling techniques, such as stratified or quota-based sampling, to ensure broader inclusion and improve generalizability. A similar sampling pattern was observed in previous Palestinian research using online surveys (10).

Additionally, the exclusive use of quantitative methods limits the ability to capture the emotional, cultural, and contextual nuances of public perceptions. Beliefs surrounding epilepsy particularly those rooted in fear, spiritual interpretations, or personal experiences may not be fully reflected in structured survey responses. Future research should consider mixed-methods designs that incorporate qualitative components such as focus groups or in-depth interviews. These can provide deeper insights into how individuals interpret epilepsy, respond to stigma, and navigate community-based support systems.

Future research should address these gaps by employing community-based sampling to include underrepresented groups such as older adults and rural populations. Mixed-methods approaches incorporating qualitative interviews or focus groups could uncover the nuanced beliefs and experiences that shape public perceptions of epilepsy. Additionally, longitudinal studies are recommended to evaluate the effectiveness of public education campaigns and to monitor changes in attitudes and knowledge over time.

Moreover, Healthcare system limitations also influence public understanding. In countries like Lebanon and China, better-organized health sectors and integrated public health campaigns have fostered greater epilepsy literacy. In contrast, the Palestinian context, characterized by political instability, restricted healthcare access, and limited funding for long-term awareness programs, likely contributes to the observed gaps in knowledge and awareness. Nonetheless, the high rate of positive attitudes indicates a societal readiness to support individuals with epilepsy if provided with accurate information and resources.

The observed gap between moderate knowledge and strong positive attitudes presents a unique public health opportunity. Positive social attitudes provide a fertile ground for targeted educational interventions aimed at correcting misconceptions and improving practical knowledge, particularly regarding first-aid measures and treatment adherence. Public health efforts should prioritize mass media campaigns, school-based health education, and community engagement through religious and local leaders. Supporting families of individuals with epilepsy through counseling and peer networks can further reduce stigma and promote inclusion.

## Conclusion

This study highlights a complex but hopeful picture of epilepsy perceptions in Palestine. While knowledge and awareness remain moderate and marked by specific misconceptions, public attitudes toward individuals with epilepsy are largely positive and inclusive. Education, particularly at higher levels, stands out as the strongest predictor of improved understanding and supportive attitudes. These findings underscore the critical need for well-designed, culturally sensitive educational interventions that target gaps in knowledge and address enduring myths. Leveraging existing positive attitudes through targeted health education and media outreach can pave the way for greater social inclusion and better health outcomes for people with epilepsy. By building on this supportive societal foundation, future public health initiatives can drive meaningful change in how epilepsy is understood and managed in Palestinian communities.

## Data Availability

The raw data supporting the conclusions of this article will be made available by the authors, without undue reservation.
